# Comparison between concurrent and sequential chemoradiation for non-small cell lung cancer *in vitro*

**DOI:** 10.3892/ol.2013.1707

**Published:** 2013-11-26

**Authors:** SEO-YOUNG SONG, AMIT K. DAS, JOHN D. MINNA

**Affiliations:** 1 Department of Internal Medicine, Kangwon National University Hospital, Chuncheon-si, Gangwon-do 200-722, Republic of Korea; 2 Hamon Cancer Center for Therapeutic Oncology and Simmons Cancer Center, University of Texas Southwestern Medical Center, Dallas, TX 75390, USA

**Keywords:** chemotherapy, radiation, concurrent, sequential, carcinoma, non-small cell lung cancer, cell line

## Abstract

Current practice guidelines recommend the combination of chemotherapy and thoracic radiation for locally advanced non-small cell lung cancer (NSCLC). Previous meta-analyses have shown that concurrent chemoradiation (CCRT) may be superior to sequential chemoradiation (SCRT). However, few previous *in vitro* studies have analyzed these two treatment schedules. In the current study, four lung cancer cell lines harboring wild-type epidermal growth factor receptor, comprising two squamous and two non-squamous cell lines, were used. The IC_10_ concentrations of three platinum-based regimens were combined with radiation treatment. Cells were irradiated at 0, 2, 4, 6 and 8 Gy using a ^137^Cs irradiator concurrently or sequentially. Surviving fractions (SFs) were plotted as a function of the radiation dose. In A549 cells, only the docetaxel (Doc) and carboplatin (Carbo) combination showed a significant radiosensitizing effect with CCRT treatment. For the other three cell lines, no difference was identified in the SFs between CCRT and SCRT. An *in vitro* method of comparing CCRT with SCRT was established using lung cancer cell lines. Overall, no significant difference was detected in the radiosensitizing effect of the two treatment schedules, with the exception of the A549 cell lines treated with Doc/Carbo.

## Introduction

Lung cancer is the most common cause of cancer-related mortality worldwide (1). Non-small cell lung cancer (NSCLC) accounts for 80% of all types of lung cancer, and up to one-third of all patients with NSCLC present with locally advanced disease that is surgically unresectable. Current practice guidelines recommend that these cases be treated with a combination of chemotherapy and thoracic radiation (2). The two used methods of combining these two modalities are concurrent chemoradiation (CCRT), defined as chemotherapy administered on the same day as radiotherapy, and sequential chemoradiation (SCRT), usually administered as two to four cycles of chemotherapy prior to radiotherapy. The NSCLC Collaborative Group performed a meta-analysis of six randomized trials that compared the outcomes of SCRT versus CCRT (3). The chemotherapy regimens varied among the trials, but all incorporated a platinum-based agent. The meta-analysis showed that CCRT yielded improved local control and median survival rates, possibly due to its radiosensitizing effect. However, CCRT has been associated with more toxic adverse events, particularly treatment-related mortality and acute esophagitis. Furthermore, previous trials have exhibited poor patient accrual and have been terminated prematurely, therefore, the statistical power of these studies has been insufficient to detect a significant benefit. Thus, certain authors have insisted that future studies are required to support CCRT as the standard of care (4).

Compared with clinical data, little *in vitro* data concerning these two treatment schedules are available. The present study conducted *in vitro* chemoradiation using NSCLC cell lines to detect differences in chemoradiosensitivity according to the treatment schedule used.

## Materials and methods

### Cell culture

Four NSCLC cell lines, A549 (adenocarcinoma), H1299 (large cell neuroendocrine carcinoma), HCC15 [squamous cell carcinoma (SQCC)] and H157 (SQCC) were obtained from the Hamon Cancer Center for Therapeutic Oncology Research, University of Texas Southwestern Medical Center (Dallas, TX, USA). These cell lines were maintained at 37°C and 5% CO_2_ in RPMI 1640 with glutamine (Hyclone, Logan, UT, USA) containing 5% fetal bovine serum (FBS; Gemini Bio Products, West Sacramento, CA, USA).

### Colony formation assay

The following three platinum-based regimens were used: Gemcitabine (Gem)/cisplatin (Cis), pemetrexed (Pem)/Cis and docetaxel (Doc)/carboplatin (Carbo). For chemotherapy combined with radiation, the IC_10_ values of these three platinum-based regimens were determined using colony formation assays. The results are presented as the IC_10_ values of Gem, Pem and Doc in each combination. The IC_10_ values of Cis and Carbo were then calculated using the drug ratio based on the doses used in the clinic.

### Clonogenic cell survival assay

**Figure 1:**
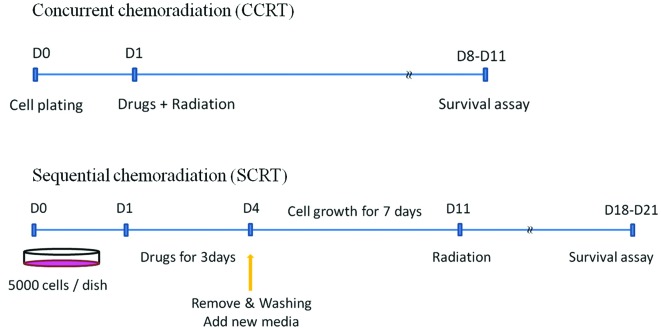
Treatment scheme for concurrent chemoradiation (CTRT) and SCRT *in vitro.* D, day.

Fig. 1 shows the *in vitro* chemoradiation treatment scheme. For CCRT, the cells were seeded in triplicate six-well plates containing RPMI 1640 plus 10% FBS at various densities commensurate with the dose of radiation. These cells were treated with chemotherapy at the IC_10_ and with radiotherapy on the same day. For SCRT, the cells were plated on 100-mm dishes and treated with chemotherapy at the IC_10_ on day 1 (D1). To terminate drug exposure, the medium was removed and the dishes were then washed twice with medium on D4. The cells were allowed to grow for seven days and then irradiated on D11 following replating on six-well plates.

The cells were irradiated at various doses (0, 2, 4, 6 and 8 Gy) using a ^137^Cs irradiator (Mark I-68; J. L. Shepherd and Associates, San Fernando, CA, USA). Colonies containing >50 cells were stained with crystal violet and manually counted using a microscope. The percentage cell viability of the irradiated samples [shown as plating efficiency (PE)] was calculated as the fraction of cell viability relative to 100% viability of the untreated samples and plotted as a function of the radiation dose. Surviving fractions (SFs) were plotted as a function of the radiation dose. Cell survival curves were generated using the following multitarget, single-hit cell survival equation:

$$S=1-{\left(1-e^{-D/D_{0}}\right)}^{n}$$

S indicates the SF at dose D, while D_0_ indicates the dose required to reduce the SF to 37% and n indicates the total number of targets at 0 Gy. The radiation sensitivity was expressed as the SF at 2 Gy (SF2).

## Results

**Figure 2:**
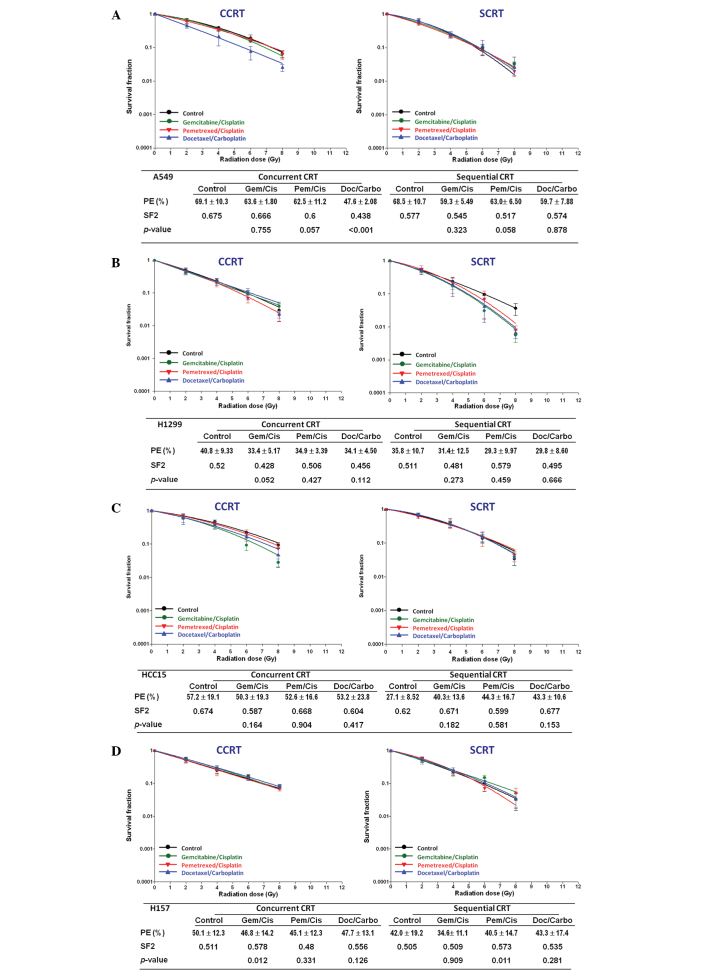
Results of the chemoradiation for each cell line; (A) A549, (B) H1299, (C) HCC15 and (D) H157. CCRT, concurrent chemoradiation; SCRT, sequential chemoradiation; Gem; gemcitabine; Cis, cisplatin; Pem, pemetrexed; Doc, docetaxel; Carbo, carboplatin; PE, plating efficiency, SF2, surviving fraction at 2 Gy.

Table I shows the IC_10_ values for each doublet. To these concentrations, radiation was then added concurrently or sequentially. In the H1299, HCC15 and H157 cells, no differences were identified between CCRT and SCRT (Fig. 2B–D). For the HCC15 cells, even if the PE of the control in the sequential arm was lower than others, no significant decrease was identified in SF2 value. In the A549 cells, only the Doc/Carbo combination showed a significant radiosensitizing effect in CCRT (Fig. 2A).

## Discussion

In locally advanced NSCLC, combined-modality chemoradiation is usually used in current practice. CCRT is likely to be superior to SCRT according to the results of several previous studies (2,5,6). However, CCRT is known to be more toxic in terms of esophagitis and pneumonitis. The effect of the radiation schedule *in vitro* is not known, since no data exists. The current study conducted chemoradiation concurrently or sequentially to determine the effects of the three platinum doublets in lung cancer cell lines. Platinum-based chemotherapy regimens are used widely, but to date, no consensus has been reached with regard to the optimal chemotherapeutic regimen. The present study assessed three doublets that are widely used in clinical practice. The cytotoxicity of an agent is usually expressed as the IC_50_ value. However, the IC_10_ was used since the majority of cells were unable to survive treatment with the IC_50_ concentration when combined with radiation.

The four cell lines used in the current study harbored wild-type epidermal growth factor receptor (*EGFR)*. Das *et al* identified a subset of naturally occurring *EGFR* mutations that lack a critical radioprotective function of EGFR (7). In the future, additional studies using more cell lines must compare the two treatment schedules according to mutational status. The methods used in the current study are likely to facilitate conducting such *in vitro* studies of combination modalities.

In conclusion, the present study established an *in vitro* method of comparing CCRT with SCRT using lung cancer cell lines. Overall, no significant difference was detected in the radiosensitizing effect of the two treatment schedules, with the exception of the A549 cell line treated with Doc/Carbo. The underlying mechanism should be elucidated.

## Figures and Tables

**Table I:** IC_10_ values of three platinum doublets for each cell line.

	Gem/Cis, nM/nM		Pem/Cis, μm/μm		Doc/Carbo, nM/nM	
			
Cell line	Gem	Cis	Pem	Cis	Doc	Carbo
A549	0.486	0.034	0.412	0.123	0.127	1.476
H1299	0.170	0.119	0.040	0.012	0.038	0.443
HCC15	0.922	0.065	0.126	0.028	0.030	0.346
H157	0.198	0.014	0.121	0.036	0.070	0.810

Gem; gemcitabine; Cis, cisplatin; Pem, pemetrexed; Doc, docetaxel; Carbo, carboplatin.
